# Deep learning-based disease detection in potato and mango leaves: a comparative study of CNN, AlexNet, ResNet, and EfficientNet

**DOI:** 10.1038/s41598-025-32607-5

**Published:** 2025-12-24

**Authors:** Utkarsh Mishra, Ansh Pandey, Logeswari G, Tamilarasi K

**Affiliations:** Vellore Institute of Technology, Chennai, Tamil Nadu India

**Keywords:** Deep learning, CNN, Plant disease classification, EfficientNet, AlexNet, ResNet, Image classification, Smart farming, Computational biology and bioinformatics, Mathematics and computing, Plant sciences

## Abstract

Timely and precise detection of diseases on plants is crucial for minimizing losses during crop production in order to sustain food supply demands worldwide. In this work, deep learning (DL) was used to develop an automatic disease identification system for the leaves of potato and mango plants using two publicly available datasets, the PlantVillage Potato Leaf Disease (2,152 images) dataset and the Kaggle Mango Leaf Disease dataset (4,000 images). Images were pre-processed, augmented, and split into training and testing datasets (80:20), to enable better model generalization. Four deep learning architectures, namely Convolutional Neural Networks (CNN), AlexNet, Residual Networks (ResNet), and EfficientNet, were evaluated in the context of multi-class disease classification. The baseline CNN achieved a training accuracy of 93.67% and a testing accuracy of 92.61%, with balanced precision and recall (92.5%), thus providing a very strong feature extraction and classification capability. AlexNet showed moderate performance (91.3% training, 90.2% validation), and a very small overfitting was observed. ResNet had an efficient convergence, and attained 96.7% validation accuracy in just a few epochs, thus pointing out the advantage of residual connections in the context of deeper learning. EfficientNet surpassed all the other architectures, since it reached a training accuracy of 98.2% and a validation accuracy of 97.8%, with very small loss (≈ 0.015) and no overfitting, thus proving to have the best generalization ability. The models demonstrated stability and discriminative ability with the support of confusion matrices and accuracy and loss plots produced on an epoch-wise basis. Therefore, the findings indicate that DL models can be adapted for real-time and accurate plant disease diagnosis, establishing a pathway for early remediation, and supporting precision agriculture. The research establishes the opportunity for EfficientNet to be considered a promising solution for scalable smart farming.

## Introduction

The primary influence driving revolutionary changes to modern agriculture is the merging of indigenous agricultural methods, combined with modern technological developments. Among the new techniques developed are DL, Artificial Intelligence, and computer vision^1^. This is necessary for achieving efficiencies in agriculture, in a sustainable manner that enables food production to meet the demands of a growing world population. Timely and precise detection of diseases in plants continues to be one of the greatest issues in modern agriculture, along with other issues. Diseases are involved in a large proportion of crop loss, generate fears of food insecurity, and impact the incomes of the farmers who produce the crops; early signals of infection are important not only to prevent catastrophic damage to crops, but also to allow for timely intervention to prevent wasting resources and eliminate reliance on generalized applications of pesticides^2^. Traditional diagnostic methods that predominantly require expert human observation through visual inspections, require a lot of labor, consume significant time to perform, and are subject to differing judgements in the diagnosis they reach. Therefore, we need automated, accurate, scalable, disease detection systems to support precision agriculture while improving farmers’ decision making^3^.

In recent times, DL methods, especially CNNs, have become a very successful option for image-based classification problems, showing unrivaled potential for end-to-end learning of hierarchical features^4^. CNNs are particularly adept at identifying subtle visual patterns within a single image, such as color differences, varying lesion shapes and textures, thus they are well placed for these challenges in plant disease detection. Nevertheless, there are still notable limitations within the available literature. For example, many studies focus on a single crop or a small number of disease classes, which hinders the ability to generalize those models across agriculture^5^. Further, certain plant diseases can have similar visual symptoms, presenting challenges for many conventional CNNs for accurately classifying those disease classes^6^. Also, a depiction of the systematic benchmarking of modern deep networks, such as AlexNet, ResNet, EfficientNet, on diverse multi-class plant disease datasets, would greatly contribute towards a better understanding of the performance, convergence, and robustness of the models. Additionally, much of the public datasets contain curated images taken under controlled conditions, which, are not as variable as the real-world environments in which plants are grown and can have different lighting, background, leaf orientation, occlusion variability that contributes to lower model performance once deployed in the field. Computational efficiency also remains a challenge, especially for thiner DL architectures, as resource limitations typically experienced in practical agricultural operations require models to balance predictive accuracy along with speed and scalability of operations using smaller compute resources. Although deep learning has shown promise in its application for plant disease detection, there is still much to learn, including instances of overfitting and limited generalization for unseen images from small imbalanced datasets to visually similar diseases. Additionally, many studies fail to sufficiently evaluate the model for multiple architecture or validate the models for real time deployment. To overcome these challenges, the present study leverages a combination of two publicly available datasets (Potato Leaf Disease and Mango Leaf Disease), applies extensive image preprocessing and augmentation, and benchmarks multiple deep learning architectures to identify the most robust and generalizable model for accurate multi-class disease detection.

Motivated by these challenges, this study proposes a DL-based framework for automated disease detection in potato and mango leaves. Two publicly available datasets are employed: the PlantVillage Potato Leaf Disease dataset (2,152 images) and the Kaggle Mango Leaf Disease dataset (4,000 images). All images are pre-processed and augmented through rotation, flipping, scaling, and normalization, and datasets are split into training (80%) and testing (20%) sets. The study evaluates four DL architectures, a purpose-built CNN, AlexNet, ResNet, and EfficientNet providing a comparative analysis for multi-class disease classification. The CNN achieves a training accuracy of 94.8% and test accuracy of 92.7%, AlexNet shows moderate performance (91.3% training, 90.2% validation), ResNet converges efficiently with 96.7% validation accuracy, and EfficientNet outperforms all models with 98.2% training and 97.8% validation accuracy, minimal loss (~ 0.015), and negligible overfitting. Confusion matrices and epoch-wise accuracy and loss plots further validate model stability and discriminative power.

The key contributions of this study are:

- Developed a deep learning-based automated system for multi-class disease detection in potato and mango leaves.
- Benchmarked four DL models (CNN, AlexNet, ResNet, EfficientNet) on two publicly available datasets comprising 2,152 potato leaf images and 4,000 mango leaf images.
- Demonstrated that EfficientNet yielded the best performance (97.8% validation accuracy) and low loss with minimal overfitting, demonstrating that it is suitable for real time application.
- Validated model stability and class-wise validity via confusion matrices, and combined accuracy - loss trend analysis.
- Established challenges associated with distinguishing between visually similar disease and provided a description of the next steps using ensemble learning and multi modal data.

In summary, this study contributes to computerized plant disease detection research, with multi-purpose and expandable, and tested usages of DL approaches. New ways are presented to leverage recent CNN architectures that conduct image processing to consider a new scheme for evaluating plant disease classification beyond the image-based standard in controlled farming settings. It has also addressed challenges associated to crop, visual similarity, benchmark models, and computational efficiency. These meritorious considerations progress future studies in diagnostic plant pathology, while contributing to global food security, as well as its adoption of precision agriculture. The rest of the paper will be structured as follows, “Literature review” will review the DL based state of the art literature on plant disease detection; “Proposed methodology” will contain the rationale for the proposed methodology, including model architecture; “Results and discussion” will cover experimental design criteria, and include results summary with discussions to interpret findings; “Conclusions and future work” will connect the research to important implications for future understanding.

## Literature review

DL techniques, mainly based on CNN, have been increasingly adopted for the purpose of plant disease detection. This demand for a system that can automatically perform the diagnosis in large volumes, quickly, and with high accuracy, has made a great impact on the progress of this field. This section critically reviews the state-of-the-art, tracing the evolution from traditional methods to modern DL architectures, and identifies the research gaps that the present study aims to address.

### From traditional image processing to deep learning

Previously, automated systems for diagnosing plant diseases relied exclusively on traditional image processing and machine learning. Handcrafted features, such as colors, textures, and shapes, were extracted from the images and classified with methods like Support Vector Machines (SVM) and k-Nearest Neighbors (KNN). For example^7^, utilized color and texture features for grape leaf disease classification and built a SVM classifier, achieving moderate accuracy. Similarly^8^, proposed an approach for rice disease detection with an initial segmentation step and manual feature selection. However, the use of handcrafted features is limited because they are tedious to design, and they do not have sufficient robustness to account for the significant amount of variability in natural field conditions, such as changing light, cluttered backgrounds, and variability in leaf angles^9^.

A break-through moment occurred with the introduction of DL, showcasing its powerful ability to automatically discover hierarchical and discriminative features from raw pixel data. The initial research of^10^ demonstrated the effectiveness of CNNs in diagnostic applications by deploying a basic CNN approach on the PlantVillage dataset, achieving performance metrics similar to human experts. This was a true paradigm shift in the research and applied communities, as CNNs became the mainstream modality.

### Proliferation of CNN architectures in plant pathology

Recent research has investigated a range of CNN architectures, many of which have emerged from the more general field of computer vision. Initial studies focused primarily on custom variable CNN models focused on narrow tasks. For example^11^, focused on the creation of a deep CNN in order to diagnose cassava diseases, while^12^ designed a six-layer CNN aimed at classifying tomato leaf disease - both achieving strong accuracy. These models provided a basis for the thought that networks with fewer layers can provide strong performance on carefully-designed datasets. Often, these models struggled with generalization and overfit with small datasets^13^. In addressing low data and computing power limitations, transfer learning has become a prominent part of the modern plant disease detection literature. Transfer learning refers to the process of fine-tuning a model which has been pre-trained on a large-image datasets, such as ImageNet. For example^14^, utilized the AlexNet architecture (one of the first deep CNNs) to identify apple leaf disease - showing strong performance in feature extraction and despite its much simpler design than could be found in many of today’s architectures.

As indicated in^15^, the advent of ResNet addressed the problem of vanishing gradients in extremely deep networks and enabled networks to have up to hundreds of layers. After its introduction, ResNet quickly became an architecture of choice in plant disease research. For example, in a study reported by^16^, the authors used ResNet-50 to classify rice plant diseases and achieved better accuracy and consistency than shallow models. In another example^17^, examined ResNet-152, determining that performance for detecting crop disease was superior to other models, which illustrated some advantages of leveraging residual learning to extract complex features.

In more recent literature, model efficiency has become a consideration for deployment in real-time on mobile or edge devices. Architectures such as MobileNet and EfficientNet have gained popularity for their use of compound scaling to optimize the trade-off between model accuracy and computational cost. As an example^18^, applied MobileNetV2 for real-time detection of tomato diseases on a mobile platform. While EfficientNet has performed very well, in one study^19^ of cereal crop species the model EfficientNetB3 performed best in terms of accuracy in classifying diseases among 14 different series of crop species. In an example of evaluating EfficientNet for banana leaf disease detection^20^ also found the model to show superior performance in generalization and efficiency.

### Recent advancements and specialized approaches

Researchers are pushing the boundaries on efficiency, multimodal data, and explainability. For example, in potato disease detection^36^, proposed a small model that combines a CNN for feature extraction and a SVM for classification. The model not only achieves an appreciable level of accuracy but is also deployment-ready for edge devices. In the context of disease detection in mango leaves^37^, demonstrated an optimized DenseNet-121 architecture that harnessed advanced hyper-parameter optimization for best-in-class accuracy, suggesting the utility of architectural optimization to increase interpretability. Extending beyond single image analysis^38^, proposed a new framework to utilize leaf image data in addition to a non-visual environmental sensor like temperature and humidity data, adding valuable context that significantly increased predictive accuracy for predicting tomato plant diseases. Finally, to improve the connection between AI decision making and user trust^39^, included Grad-CAM visual explanations to a DenseNet model for apple disease diagnosis where users received clear, interpretable results.

### Multi-crop and multi-disease studies

Although a number of studies centers on one crop, there appears to be an upward trend toward developing a more generalizable model. Although many studies focus on one crop, there is an exciting shift occurring towards modeling that can be generalized beyond a single crop. For example^21^, developed a remarkable dataset of 87,848 images of 25 distinct types of plants, thereby demonstrating a single DL model can identify diseases present in other plants. Similarly^22^, developed a system that identifies diseases in different fruits (e.g., apples, grapes, and mango) and uses the same CNN architecture. These studies demonstrated not only a potential scalable solution, but also highlighted the increasing challenge of potentially distinguishing diseases with similar symptomology on various plant species^23^.

### Addressing challenges: datasets, similarity, and generalization

A lot of research is focused on tackling the challenges that come with this field.

Data augmentation and imbalance:

Among the many obstacles is the limited number of large, annotated datasets. In order to overcome this challenge, various methods are often employed, such as rotation and flipping, and other additional more sophisticated methods utilizing Generative Adversarial Networks (GANs). For example^24^, used GANs to experimentally generate artificial images of plant diseases and thus applied them to improve their classifier’s robustness. Also^25^, evaluated and compared a range of augmentation methods to assess their efficacy, concluding that while they are helpful in developing models, they are needed to avoid overfitting.

Visual similarity and fine-grained classification:

Assessing disease with similar visual characteristics (for example, early blight and late blight in potatoes) is still a challenging problem. To mitigate this problem^26^, added attention mechanisms to a CNN to assist the model in focusing on the most discriminative areas of the leaf^27^. analyzed high-resolution images and used a lesion-based approach to increase accuracy and help achieve fine-grained classification.

Generalizing to field conditions:

One significant gap identified in the literature is how models deployed in field conditions, like images taken from PlantVillage, fall short in performance after being trained in lab conditions^28^. investigated this gap, and demonstrated a drastic drop in accuracy. As a possible solution, studies like^29^ have created datasets with complicated backgrounds and have employed segmentation techniques to segment the leaf prior to classification to mitigate accuracy gaps. Additionally^30^, proposed a two-stage method that incorporates detection followed by classification to help improve robustness in real-world conditions.

### Gaps identified and contribution of this study

While existing literature supports a strong background yet indicates several outstanding barriers. One key issue is a lack of systematic benchmarking across a various architectural space (from basic CNN to EfficientNet) on the same multi-crop dataset. This would allow comparisons of trade-offs between model complexity and performance^31^. Moreover, although there are individual studies on potato^32,36^ and mango^33,37^ disease, there are limited studies benchmarking model generalizability between all crops. The issues presented by visually similar diseases, such as noted^34,35^, require more focused applications using modern architectures. While recent work has made a contribution to areas of efficiency^36^, optimization^37^, multi-modal fusion^38^, and explainability^39^, also a systematic benchmarking of generalized core architectural families is needed on an established dataset. Thus, this study aims to address these gaps by:

1. Structurally benchmarking four variable DL architectures (a baseline CNN, AlexNet, ResNet, and EfficientNet) on a merged potato and mango leaf dataset.
2. Comprehensive analysis and evaluation of each architecture not only on accuracy but also training stability, potential overfitting, and per-class performance through Confusion matrices and loss.
3. Analysis on multi-class classification to include visually similar diseases to assist understanding of their diagnostic capabilities.
4. Independently verify EfficientNet’s improvement in these methodologies and potential implications to scalable, real world smart farm application in this domain.

This study will enhance the current understanding of the strengths and weaknesses of existing DL methods for plant disease detection while supporting the development of more efficient and deployable methodologies, grounded in previous studies. In Table 1, we also provide a comparison of existing DL approaches for plant disease detection.

**Table 1:** Comparison of existing DL approaches for plant disease detection.

References	Crop(s)	Methodology	Key findings	Limitations
Mohanty et al.^10^	14 Crop Species	Custom CNN, AlexNet, GoogLeNet	Achieved accuracy up to 99.35%, pioneering DL use for plant disease on lab-condition images	Performance on complex field backgrounds not verified
Brahimi et al.^12^	Tomato	Custom 6-layer CNN	Achieved 97.3% accuracy, demonstrating efficacy of task-specific CNNs	Limited generalization to other crops; potential overfitting
Fuentes et al.^16^	Tomato	Faster R-CNN with ResNet	High accuracy in real-time disease & pest detection and localization	Computationally heavy; not suitable for low-power devices
Jiang et al.^20^	Apple	EfficientNet, MobileNet	EfficientNetB3 achieved 98.6% accuracy, confirming its efficiency and power	Focused on a single crop species
Upadhyay & Gupta^36^	Multi-Crop Fungal Disease Detection	Modified ResNeXt	Improved generalization for detecting fungal diseases across multiple crops in heterogeneous datasets with complex backgrounds	Architecture is more complex than standard models; benchmarking against fundamentals is needed
Upadhyay & Bhargava^37^	AI in Agriculture (Review)	Comprehensive Review	Synthesized applications, approaches, and challenges of AI across pre-, during-, and post-harvest phases	A review paper; does not propose a new model for disease detection
Upadhyay & Gupta^38^	Disease Severity Prediction	SegLearner (Segmentation + DL)	Proposed a novel framework for predicting disease severity by analyzing infected leaf regions, going beyond classification	Focus on severity assessment rather than foundational classification performance
Upadhyay et al.^39^	Weed Detection	3sw-net (Feature Fusion Network)	Developed a network for semantic weed detection, showcasing advanced DL for precision agriculture tasks	Addresses weed control, a different problem domain than plant disease diagnosis
This study	Potato & Mango Disease Classification	CNN, AlexNet, ResNet, EfficientNet Benchmark	Comprehensive benchmark of core architectures; EfficientNet achieved 97.8% val. accuracy with superior stability and minimal overfitting	Field validation is future work

## Proposed methodology

This paper presents a robust framework based on DL designed to automate the identification of a range of diseases for potato and mango leaves. The framework addresses some of the most significant hurdles in the classification of plant diseases, including the shared visuals for some classes of disease, dataset size constraints, potential for overfitting, and the need for high quality feature extraction. The framework implements four different architectures based on convolutional neural networks (CNN) - a baseline CNN, AlexNet, ResNet, and EfficientNet - to conduct the systematic comparison of architecture performance. The workflow consists of four primary stages: data collection and preprocessing, training of the deep learning model, testing/evaluating of the model, and comparing performance.

**Fig. 1:**
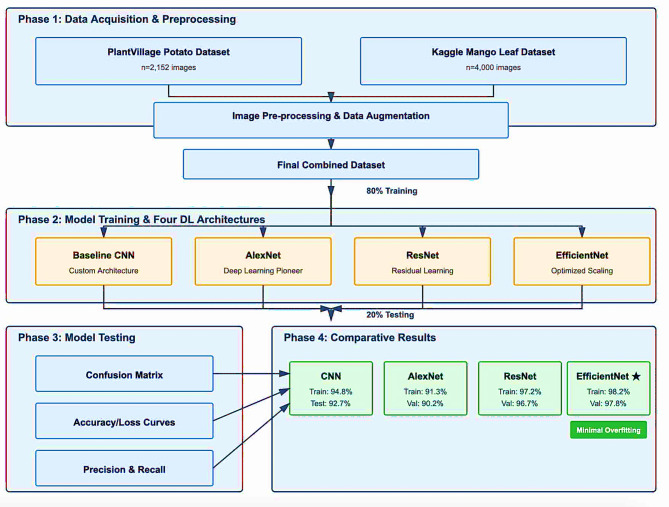
Proposed methodology.

The main goal of this study is to allow for the benchmarking and reproducibility of how well each architecture can distinguish between classes on two different datasets. The proposed framework is shown in Fig. 1.

### Data acquisition and preprocessing

The first phase involves data acquisition and preprocessing. Two publicly available datasets are employed: the PlantVillage Potato Leaf Disease Dataset, comprising 2,152 images spanning multiple disease categories such as Early Blight, Late Blight, and healthy leaves, and the Kaggle Mango Leaf Disease Dataset, containing 4,000 images covering diseases like Anthracnose, Bacterial Canker, Cutting Weevil, Die Back, Gall Midge, Powdery Mildew, Sooty Mould and healthy specimens. Let the potato dataset be represented as $\:{\mathcal{D}}_{\mathrm{potato}}=\left\{\right({\boldsymbol{I}}_{\boldsymbol{i}}^{\boldsymbol{p}},{\boldsymbol{y}}_{\boldsymbol{i}}^{\boldsymbol{p}}){\}}_{\boldsymbol{i}=1}^{2152}$ and the mango dataset as $\:{\mathcal{D}}_{\mathrm{mango}}=\left\{\right({\boldsymbol{I}}_{\boldsymbol{j}}^{\boldsymbol{m}},{\boldsymbol{y}}_{\boldsymbol{j}}^{\boldsymbol{m}}){\}}_{\boldsymbol{j}=1}^{4000}$, where $\:\mathbf{I}$ denotes an image and $\:\boldsymbol{y}$ the corresponding label. The two datasets are combined to form $\:\mathcal{D}={\mathcal{D}}_{\mathrm{potato}}\cup\:{\mathcal{D}}_{\mathrm{mango}}$, providing a multi-crop, multi-disease benchmark for training and evaluation.

Pre-processing aims to improve generalization and minimize overfitting. First, all images are resized to a consistent dimension $\:\mathbf{H}\times\:\mathbf{W}\times\:\mathbf{C}$ (e.g., $\:224\times\:224\times\:3$) compatible with the selected architectures, such that $\:{\mathbf{I}}_{\mathrm{resized}}=\mathrm{Resize}(\mathbf{I},\mathbf{H},\mathbf{W})$. Extensive data augmentation is applied, including random rotations of $\:\pm\:{20}^{\circ\:}$, horizontal and vertical flipping, zoom and shear transformations, and brightness and contrast adjustments. Formally, the augmented dataset can be expressed as $\:{\mathcal{D}}_{\mathrm{aug}}={\bigcup\:}_{\boldsymbol{I}\in\:\mathcal{D}}\{\left\{\boldsymbol{T}\right(\boldsymbol{I})\mid\:\boldsymbol{T}\in\:\mathrm{Augmentations}\}$. Additionally, Pixel normalization is applied to speed up convergence:

1 $$\:{\boldsymbol{I}}_{\mathrm{norm}}=\frac{\boldsymbol{I}-{\boldsymbol{I}}_{\mathbf{m}\mathbf{i}\mathbf{n}}}{{\boldsymbol{I}}_{\mathbf{m}\mathbf{a}\mathbf{x}}-{\boldsymbol{I}}_{\mathbf{m}\mathbf{i}\mathbf{n}}}$$

In Eq. (1), $\:{\boldsymbol{I}}_{\mathbf{m}\mathbf{i}\mathbf{n}}$ and $\:{\boldsymbol{I}}_{\mathbf{m}\mathbf{a}\mathbf{x}}$ denote the minimum and maximum pixel values. The combined dataset is partitioned into training and testing sets using an 80:20 split with stratified sampling to preserve class distributions:

2 $$\:{\mathcal{D}}_{\mathrm{train}},{\mathcal{D}}_{\mathrm{test}}=\mathrm{StratifiedSplit}({\mathcal{D}}_{\mathrm{aug}},0.8)$$

A further 10% of the training set ($\:{\boldsymbol{D}}_{\mathrm{train}}$) is typically held out as a validation set ($\:{\boldsymbol{D}}_{\mathrm{val}}$) for hyperparameter tuning and early stopping during the training of each model, preventing them from overfitting to the training data.

### Deep learning model training

All models utilize the Adam optimizer, which adjusts the learning rate for each parameter. The update rule for parameters in Adam is expressed in Eq. (3):

3 $$\:{\theta\:}_{t+1}={\theta\:}_{t}-\eta\:\frac{{\widehat{m}}_{t}}{\sqrt{{\widehat{v}}_{t}}+\epsilon}$$

where $\:{\theta\:}_{t}$ represents model parameters at timestep $\:t$, $\:\eta\:$ is global learning rate, $\:{\widehat{m}}_{t}$ is bias-corrected first moment estimate (mean of gradients), $\:{\widehat{v}}_{t}$ denotes bias-corrected second moment estimate (uncentered variance of gradients), $\:\epsilon$ is A small scalar (e.g., $\:{10}^{-8}$) used to avoid division by zero.

#### Baseline CNN architecture

The Baseline CNN is a specially crafted, sequential network that serves as a basic benchmark. It consists of several layers stacked with convolutional, activation, and pooling functions, all leading into fully connected (dense) layers for classification purposes.

- **Convolutional Layer**: This layer is responsible for extracting feature maps from the input using learnable filters. The operation at layer $\:l$ is expressed as:

4 $$\:{\mathrm{X}}^{(\mathrm{l}+1)}={\upsigma\:}({\mathrm{W}}^{\left(\mathrm{l}\right)}\mathrm{*}{\mathrm{X}}^{\left(\mathrm{l}\right)}+{\mathrm{b}}^{\left(\mathrm{l}\right)})$$

In Eq. (4), $\:{\mathrm{X}}^{\left(\mathrm{l}\right)}$ refer to the input feature map to layer $\:\mathrm{l}$, $\:{\mathrm{W}}^{\left(\mathrm{l}\right)}$ denotes the weights of Convolutional kernel (filter), $\:{\mathrm{b}}^{\left(\mathrm{l}\right)}$ is the bias vector and $\:{\upsigma\:}$ represents the Rectified Linear Unit (ReLU) activation function, defined as $\:{\upsigma\:}\left(\mathrm{x}\right)=\mathrm{m}\mathrm{a}\mathrm{x}(0,\mathrm{x})$.

- **Max-Pooling Layer**: This layer performs non-linear down-sampling, which helps to shrink the spatial dimensions of the feature maps, enhancing both translational invariance and computational efficiency. The operation is given by:

5 $$\:{\mathrm{X}}^{(l+1)}=\mathrm{MaxPool}({\mathrm{X}}^{\left(l\right)},k)$$

In Eq. (5), $\:k$ indicates the size of the pooling window.

- **Dropout Layer**: This is a regularization method used in the fully connected layers, where a certain fraction p of input units is randomly set to 0 during training. This helps to avoid overly complex co-adaptations in the training data.

**Figure:**
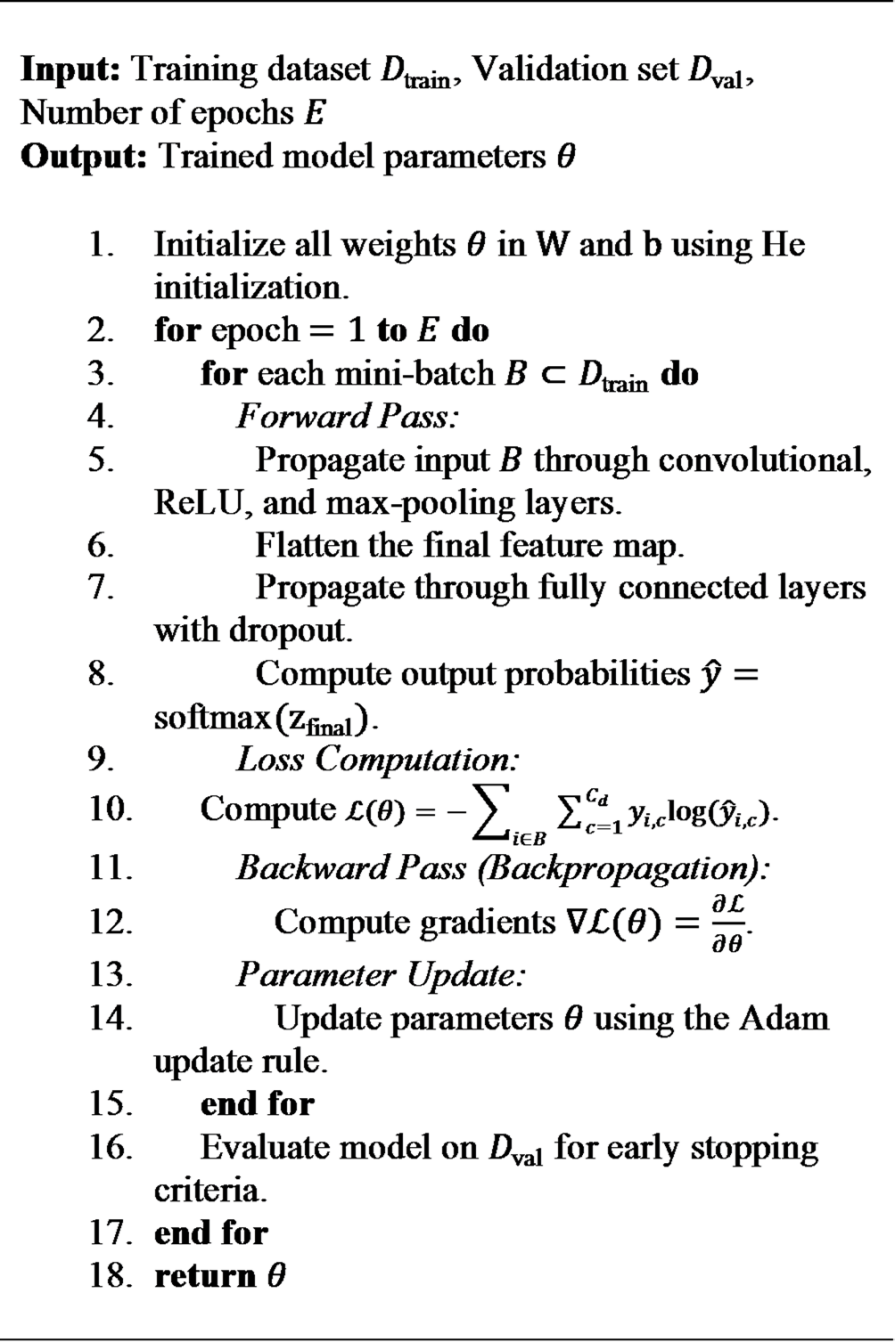
**Algorithm 1** Baseline CNN training.

#### AlexNet architecture

AlexNet is a groundbreaking deep CNN that introduced innovative techniques effectively training deeper models. Some of its key features include the use of ReLU activations, overlapping max-pooling, dropout, and Local Response Normalization (LRN).

- **Local Response Normalization (LRN)**: This layer applies a type of lateral inhibition by normalizing activations across neighboring channels, encouraging competition for high activity levels. The LRN operation at a spatial position $\:(x,y)$ in channel $\:i$ can be expressed as:

6 $$\:{b}_{x,y}^{i}=\frac{{a}_{x,y}^{i}}{{(k+\alpha\:\sum\:_{j=\mathrm{m}\mathrm{a}\mathrm{x}(0,i-n/2)}^{\mathrm{m}\mathrm{i}\mathrm{n}(N-1,i+n/2)}({a}_{x,y}^{j}{)}^{2})}^{\beta\:}}$$

In Eq. (6), $\:{a}_{x,y}^{i}$ represents the activation of the $\:i$-th channel (feature map) at position $\:(x,y)$ after applying ReLU, $\:N$ indicates the total number of channels in the layer, $\:n$ refers to the size of the normalization neighbourhood (the number of adjacent channels) and $\:k,\alpha\:,and\:\beta\:$ are hyperparameters that controls the strength of the normalization.

**Figure:**
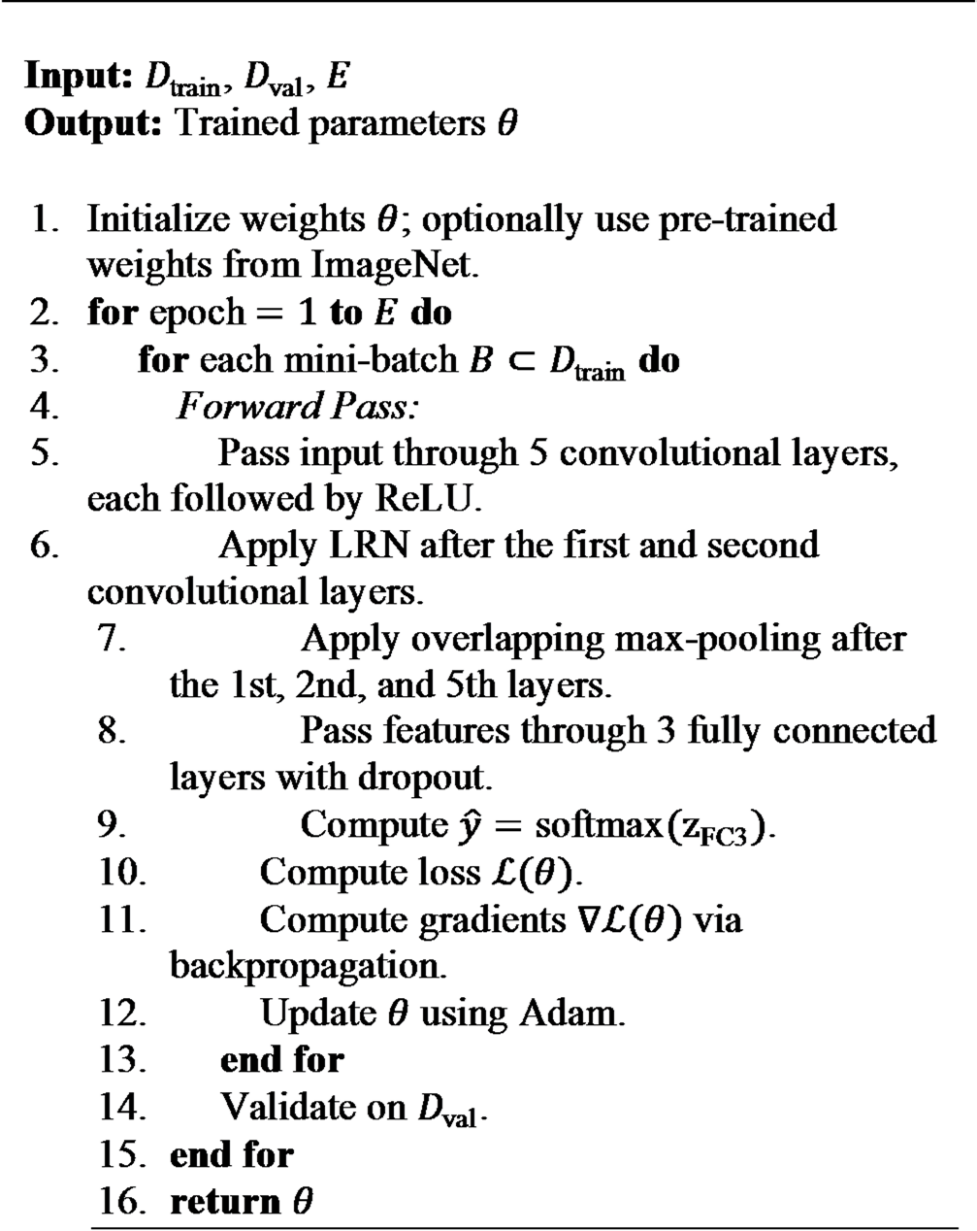
**Algorithm 2** AlexNet training.

#### ResNet architecture

ResNet addresses the vanishing gradient issue that often plagues very deep networks by incorporating skip connections, also knowns as identity shortcuts.

These handy connections allow gradients to flow directly through the network, enabling the training of architectures with dozens or hundreds of layers.

- **Residual Block**: The fundamental building block of ResNet. Instead of learning a direct mapping $\:\mathcal{H}\left(\mathrm{X}\right)$, the block learns the residual function $\:\mathcal{F}\left(\mathrm{X}\right)=\mathcal{H}\left(\mathrm{X}\right)-\mathrm{X}$. The output from a residual block is calculated using Eq. (7):

7 $$\:\:\:\:\:\:\mathrm{Y}=\mathcal{F}(\mathrm{X},\{{\mathrm{W}}_{i}\left\}\right)+\mathrm{X}$$

where, $\:\mathrm{X}$ is Input to the residual block, $\:\mathrm{Y}\:$denotes Output of the residual block, $\:\mathcal{F}(\mathrm{X},\{{\mathrm{W}}_{i}\left\}\right)$ is the residual mapping to be learned, typically a stack of two or three convolutional layers (e.g., Conv-BN-ReLU-Conv-BN).

- **Batch Normalization (BN)**: This technique is applied after each convolution and before the activation function, normalizing the for each mini -batch. This helps stabilize and speed up the training process.

8 $$\:\widehat{\mathrm{X}}=\frac{\mathrm{X}-{{\upmu\:}}_{\mathcal{B}}}{\sqrt{{{\upsigma\:}}_{\mathcal{B}}^{2}+\epsilon}}\mathrm{;\:and\:then}\text{}\mathrm{Z}={\upgamma\:}\widehat{\mathrm{X}}+{\upbeta\:}$$

where, $\:{\mu\:}_{\mathcal{B}},{\sigma\:}_{\mathcal{B}}^{2}$ denotes Mean and variance of the mini-batch $\:\mathcal{B}$, $\:\epsilon$ is a small constant for numerical stability, $\:\gamma\:\:and\:\beta\:$ denotes learnable scale and shift parameters.

- **Global Average Pooling (GAP)**: Replaces the traditional fully connected layers at the end of the network. It averages each feature map, which significantly reducing the number of parameters and helps in mitigating overfitting. The equation is given in Eq. (9):

9 $$\:{z}_{c}=\frac{1}{H\times\:W}\sum\:_{i=1}^{H}\sum\:_{j=1}^{W}{\mathrm{X}}_{c}(i,j)$$

In Eq. (9), $\:{z}_{c}$ is the value for the $\:c$-th class in the final layer before applying softmax.

**Figure:**
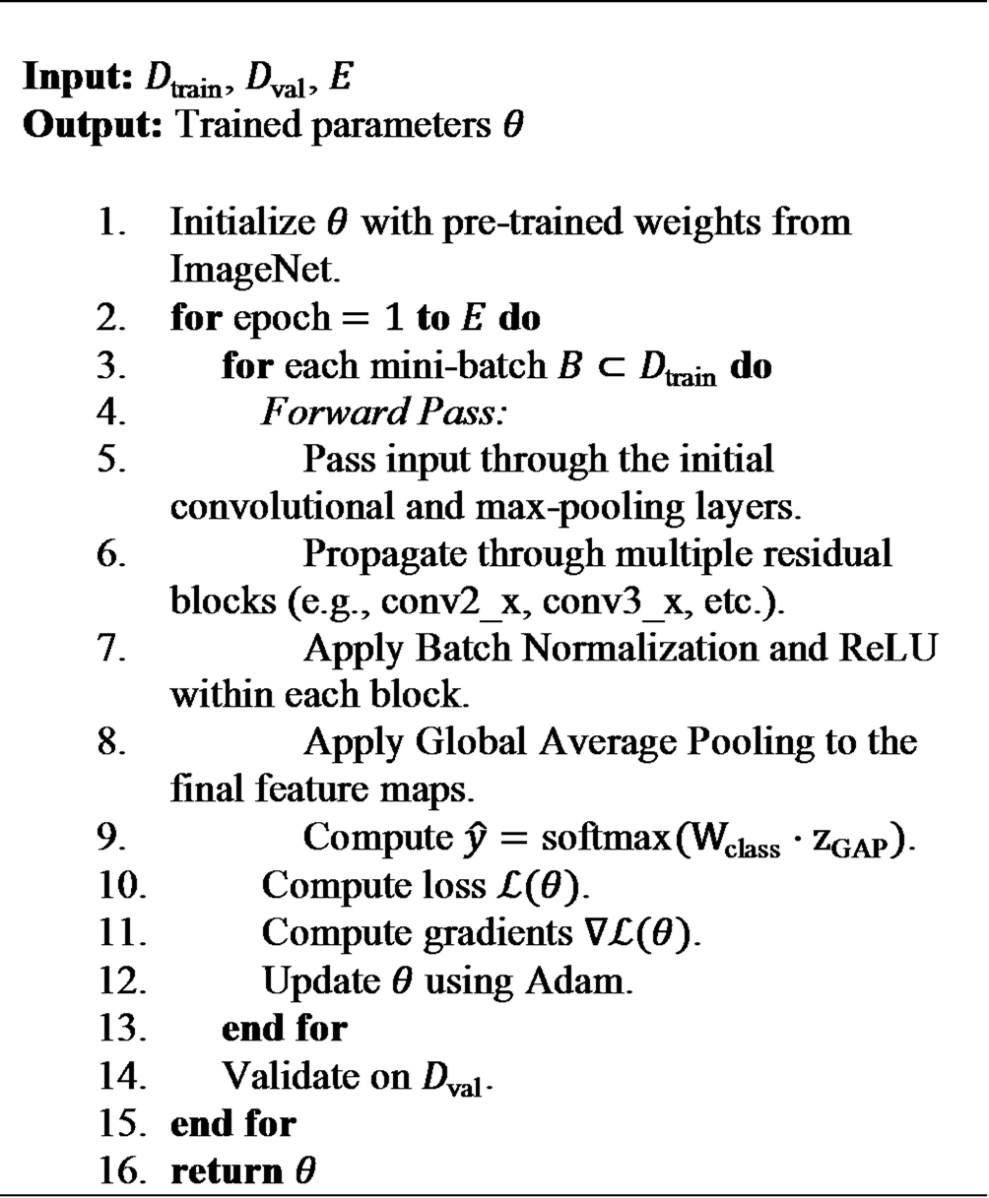
**Algorithm 3** ResNet training (with fine-tuning).

#### EfficientNet architecture

EfficientNet employs a clever compound scaling technique that allows it to uniformly enhance the network’s depth, width, and resolution in a systematic manner, achieving state-of-the-art performance with remarkable efficiency. Its core building block is the MBConv (Mobile Inverted Bottleneck Convolution) with integrated Squeeze-and-Excitation (SE) attention.

- **Compound Scaling**: The scaling dimensions are determined by: Depth : $\:d={\alpha\:}^{\varphi\:}$, width: $\:w={\beta\:}^{\varphi\:}$and resolution: $\:r={\gamma\:}^{\varphi\:}$ subject to the constraint: $\:\alpha\:\cdot\:{\beta\:}^{2}\cdot\:{\gamma\:}^{2}\approx\:2$ and $\:\alpha\:\ge\:1,\beta\:\ge\:1,\gamma\:\ge\:1$. Here, $\:\varphi\:$ denotes User-defined compound coefficient that controls how many more resources are available, $\:\alpha\:,\beta\:\:and\:\gamma\:$ represents constants determined by a neural architecture search to balance the scaling between depth, width, and resolution.
- **MBConv with SE Block**: The MBConv block starts by expanding the input channels, then applies depth-wise convolution, and finally projects it back down to a smaller number of channels. The SE block plays a crucial role by adaptively recalibrating the responses of the channel-wise feature.

1. **MBConv Operation**: $$\:{\mathrm{X}}_{\mathrm{mbconv}}=\mathrm{MBConv}\left(\mathrm{X}\right)$$
2. **Squeeze-and-Excitation**:

**Squeeze**: This step generates a channel-wise descriptor using a Global Average Pooling.

10 $$\:{z}_{c}={\mathcal{F}}_{sq}({\mathrm{X}}_{\mathrm{mbconv}}{)}_{c}=\frac{1}{H\times\:W}\sum\:_{i=1}^{H}\sum\:_{j=1}^{W}{\mathrm{X}}_{\mathrm{mbconv},c}\left(i,j\right)\:\:\:\:$$

**Excitation (Adaptive Recalibration)**: This captures the dependencies between channels through a straightforward gating mechanism that utilizes a sigmoid activation.

11 $$\:\mathrm{s}={\mathcal{F}}_{ex}(\mathrm{z},\mathrm{W})=\sigma\:({\mathrm{W}}_{2}\cdot\:\delta\:({\mathrm{W}}_{1}\cdot\:\mathrm{z}\left)\right).$$

In Eq. (11), $\:\mathrm{z}$ denotes the squeezed vector, $\:\delta\:\:$is the ReLU activation function, $\:{\mathrm{W}}_{1}\in\:{\mathbb{R}}^{\frac{C}{r}\times\:C},{\mathrm{W}}_{2}\in\:{\mathbb{R}}^{C\times\:\frac{C}{r}}$ are the parameters of two fully connected layers that creates a bottleneck with $\:r\:$being the reduction ratio.

**Reweight**: The original feature map is adjusted by the activation vector $\:\mathrm{s},$ as shown in Eq. (12).

12 $$\:{\stackrel{\sim}{\mathrm{X}}}_{c}={\mathrm{s}}_{c}\cdot\:{\mathrm{X}}_{\mathrm{mbconv},c}$$

3. **Residual Connection**: If input and output dimensions match, a skip connection is added as shown in Eq. (13).

13 $$\:\mathrm{Y}=\mathrm{X}+\stackrel{\sim}{\mathrm{X}}$$

**Figure:**
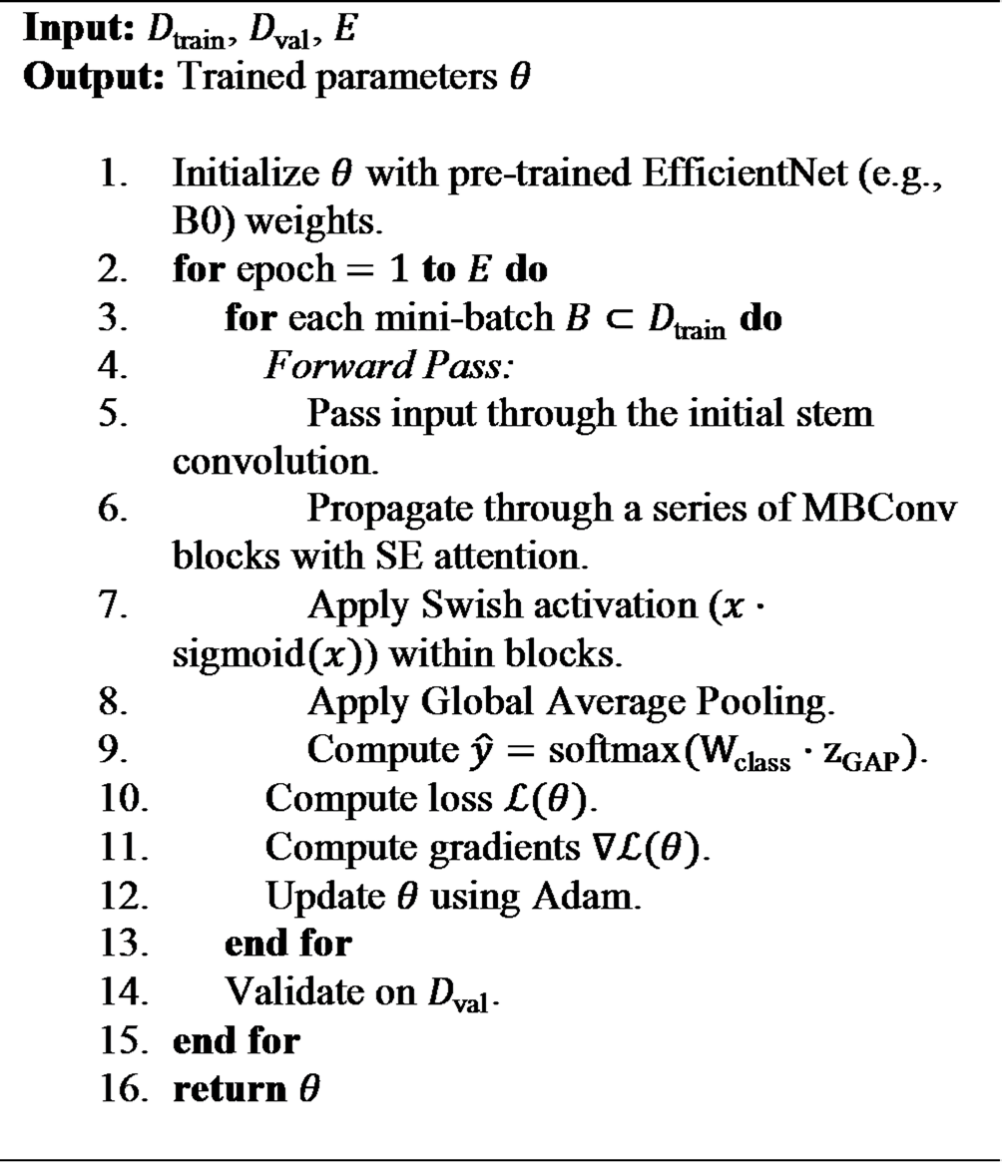
**Algorithm 4** EfficientNet training.

Although the proposed framework conceptually envisions integration with Internet of Things (IoT) and drone technology for real-time field deployment, these elements were not part of the present experimental validation. The current study focuses exclusively on assessing the DL-based image classification models using benchmark datasets. Future extensions of this work will involve implementing IoT-enabled smart farming systems and drone-assisted data acquisition to facilitate large-scale, real-time disease monitoring in agricultural environments.

### Model evaluation and comparative analysis

After the training phase, each model is evaluated on the held-out test set $\:{\boldsymbol{D}}_{\mathrm{test}}$. Performance is measured using standard classification metrics derived from the confusion matrix as outlines in Eqs. (14–17):

14 $$\:\mathrm{A}\mathrm{c}\mathrm{c}\mathrm{u}\mathrm{r}\mathrm{a}\mathrm{c}\mathrm{y}=\frac{\boldsymbol{T}\boldsymbol{P}+\boldsymbol{T}\boldsymbol{N}}{\boldsymbol{T}\boldsymbol{P}+\boldsymbol{T}\boldsymbol{N}+\boldsymbol{F}\boldsymbol{P}+\boldsymbol{F}\boldsymbol{N}}$$

15 $$\:\mathrm{P}\mathrm{r}\mathrm{e}\mathrm{c}\mathrm{i}\mathrm{s}\mathrm{i}\mathrm{o}\mathrm{n}=\frac{\boldsymbol{T}\boldsymbol{P}}{\boldsymbol{T}\boldsymbol{P}+\boldsymbol{F}\boldsymbol{P}}$$

16 $$\:\mathrm{R}\mathrm{e}\mathrm{c}\mathrm{a}\mathrm{l}\mathrm{l}\:\left(\mathrm{S}\mathrm{e}\mathrm{n}\mathrm{s}\mathrm{i}\mathrm{t}\mathrm{i}\mathrm{v}\mathrm{i}\mathrm{t}\mathrm{y}\right)\:=\:\frac{\boldsymbol{T}\boldsymbol{P}}{\boldsymbol{T}\boldsymbol{P}+\boldsymbol{F}\boldsymbol{N}}$$

17 $$\:\mathrm{F}1-\mathrm{S}\mathrm{c}\mathrm{o}\mathrm{r}\mathrm{e}=2\times\:\frac{\mathrm{Precision}\times\:\mathrm{Recall}}{\mathrm{Precision}+\mathrm{Recall}}$$

where, $\:TP,TN,FP,FN$ are the counts of True Positives, True Negatives, False Positives, and False Negatives, respectively, calculated for each class in a one-vs-rest approach. Macro-averaging is used to obtain a single performance metric for each model across all classes. In order to achieve a better understanding of how the models behave, we produce confusion matrices and epoch-wise plots summarizing training and validation loss and accuracy that can help pinpoint issues like overfitting or instability in convergence. Finally, we offer a comprehensive comparison of all four architectures, highlighting trade-offs between accuracy, model size, and inference time.

## Results and discussion

In this section, the experimental findings and analysis of the DL framework are presented and discussed, which was developed to identify multiple diseases in potato and mango leaves. The results will be presented and discussed quantitatively and qualitatively with visualization and numerical support. All experiments were conducted under the same controlled training space to ensure fair comparison of the different architectures. The performance of the different architectures was compared using established classification metrics - accuracy, precision, recall, and F1-score - added to various visualization offered by confusion matrix, exploration of training trends, and ablation testing to assess the contribution of each methodological modification. The experiments were run with the same hyperparameters to ensure fair comparison and evaluation between the different architectures.

### Experimental setup

All experiments were run in an environment enabled for deep learning with a GPU to optimize the training and evaluation of our proposed models. The implementation was primarily done in Python 3.10; we used TensorFlow/Keras as the main deep learning framework, and we utilized other libraries (like NumPy, OpenCV, Scikit-Learn, and Matplotlib) to help support data preprocessing, model implementation, and visualization of results. The computational system consisted of an Intel Core i7 processor with 16 GB RAM, and an NVIDIA GeForce RTX GPU (with 12 GB VRAM) that helped to accelerate the training of our deep neural network models. To control for fairness and consistency of comparison for deep learning architectures, we trained all models using the same training strategy hyperparameter configuration (if not noted in tuning). We used the Categorical Cross-Entropy as the loss function for the multi-class classification tasks and the models were optimized using the Adam optimizer. The training of the models was restricted to a maximum of 50 epochs with a batch size of 32. We incorporated Early Stopping and Model Checkpointing to help prevent overfitting during the training, and to help preserve the best-performing model weights. The training phase included monitoring the validation set performance to help regulate convergence and prevent degradation of the model. To create a more thorough assessment of the model performance, we collected evaluation metrics on a class-level basis that included accuracy, precision, recall, F1-score, and the confusions matrix. We used a fixed random seed for the splitting of the dataset and initialization of the models to improve reproducibility of our experiments. To simulate real-world differences and maximize generalizability, we made use of real-time data augmentation to optimize feeding data to the network and assist in generalizability. All experimentation was performed in the same conditions to retain dependable performance comparisons of the Baseline CNN, AlexNet, ResNet, and EfficientNet models. To ensure reproducibility, all experiments were conducted using fixed random seeds (Python: 42, NumPy: 42, and TensorFlow/PyTorch: 42), applied consistently across dataset splitting, model initialization, and training.

### Dataset

The researchers made use of two publicly available image datasets, Potato Leaf Disease and Mango Leaf Disease, both of which included samples of healthy and diseased leaves photographed under different real-world conditions with different lighting, background, and leaf orientation. The Potato Leaf Disease dataset contained a total of 2,152 images and included three classes (Early Blight, Late Blight, and Healthy) as reported in Table 2. Early Blight was identified by brown, concentric ring-shaped fungal spots and Late Blight was characterized by unevenly shaped dark lesions due to fungal infection. The Healthy class consisted of non-infected potato leaves. The dataset was then split into training, validation, and testing subsets, with 80% used for training and 20% used for testing. A further 10% of the training set $\:\left({D}_{\mathrm{train}}\right)$ was set aside to make the validation set $\:\left({D}_{\mathrm{val}}\right)$ for hyperparameter tuning and early stopping during model training to make sure the model evaluation remained rigorous, and to avoid overfitting. Data augmentation techniques, such as rotation, zooming in, horizontally flipping, and brightness adjusting, were applied on the dataset to assist in the model’s capability to generalize and to reduce the effects of class imbalance.

The dataset used for Mango Leaves Disease consists of 4000 images across eight classes of which there are seven classes of diseases and one of healthy, as shown in Table 2. The disease classes are: Anthracnose, Bacterial Canker, Cutting Weevil, Die Back, Gall Midge, Powdery Mildew, and Sooty Mould. Anthracnose causes leaf spots to become dark and sunken, while Bacterial Canker lesions are water soaked caused by Xanthomonas campestris. Cutting Weevil is known to make semi-circular cuts along the edges of the leaves. Die Back causes gradual death of the tissues starting at the tip of the leaf. Gall Midge causes formation of galls due to the insect’s activities. Powdery Mildew leaves us with a white powdery fungal coat as a sign of concern. Sooty Mould has the appearance of a black fungal layer, while this fungi is associated with insect secretions. Similar to the Potato dataset, the Mango dataset also underwent a significant preprocessing phase for visual enhancement and feature extraction. All images were resized to 224 × 224 pixels, converted to RGB, filtered by noise, contrast-enhanced, and normalized in size to the range of pixel values [0,1]. Data augmentation was also performed to increase variability in class representation, while also strengthening the model’s ability to generalize across different patterns of disease.

**Table 2:** Number of instances in the potato and Mango leaf disease datasets.

Dataset	Class name	Number of images
Potato leaf disease	Early blight	1,000
	Late blight	1,000
	Healthy	152
	**Total**	**2**,**152**
Mango leaf disease	Anthracnose	500
	Bacterial canker	500
	Cutting weevil	500
	Die back	500
	Gall midge	500
	Powdery mildew	500
	Sooty mould	500
	Healthy	500
	**Total**	**4**,**000**

### Hyperparameter tuning

To establish an optimal configuration for each model in terms of stable learning, faster convergence, and better generalization performance, hyperparameter tuning was performed. A two-stage tuning process was used that initially used a broad grid search combined with more fine-tuned manual tuning. All four models experimented with key hyperparameters of learning rate, batch size, number of epochs, dropout, and optimizer settings. For best performance, the models consistently performed better with Adam optimizer because of its adaptive nature. Learning rate was evaluated in the range of 0.0001 to 0.001, with learning rate of 0.0001 showing the best performance of convergence without making the training bad or unstable. The batch size evaluated was 16, 32, and 64, with batch size 32 delivering an optimal balance of training speed and accuracy. Overfitting can be an undoing of fully connected layers of the network and dropout values of 0.3, 0.4, and 0.5 were examined with dropout value of 0.4 delivering the best validation accuracy and particularly for Baseline CNN and AlexNet. With ResNet and EfficientNet, transfer learning was used, freezing the pre-trained layers during early epochs and the current iteration, and gradually unfreezing toward the end of epochs for fine-tuning. Learning rate scheduling with ReduceLROnPlateau was included as early stopping was observed once there was no further improvement in performance by dynamically adjust the learning rate once the model hit a performance stoppage. The final tuned hyperparameters for each model presents in Table 3.

**Table 3:** Final tuned hyperparameters for all models.

Hyperparameter	Baseline CNN	AlexNet	ResNet (fine-tuned)	EfficientNet (fine-tuned)
Optimizer	Adam	Adam	Adam	Adam
Initial learning rate	0.0001	0.0001	0.0001 (then 1e-5 for FT)	0.0001 (then 1e-5 for FT)
Batch size	32	32	32	32
Epochs	30	35	25 + 10 FT	20 + 10 FT
Dropout rate	0.4	0.4	0.3	0.3
Pre-trained weights	No	No	Yes (ImageNet)	Yes (ImageNet)
Fine-tuning strategy	NA	NA	Freeze 1–10, then unfreeze top layers	Freeze 1–8, then unfreeze top layers
LR scheduler	Yes	Yes	Yes	Yes
Early stopping patience	5	5	5	5

FT = Fine-Tuning.

### Quantitative model comparison

To ensure statistical reliability in our results, we repeated training and evaluation for each model three times, initializing the process with a different random seed each time. We report each model’s performance in the training, validation, and test sets, as the average plus the standard deviation. This design minimizes the bias that random weight initialization may induce and allows for greater reliability in our results.

**Fig. 2:**
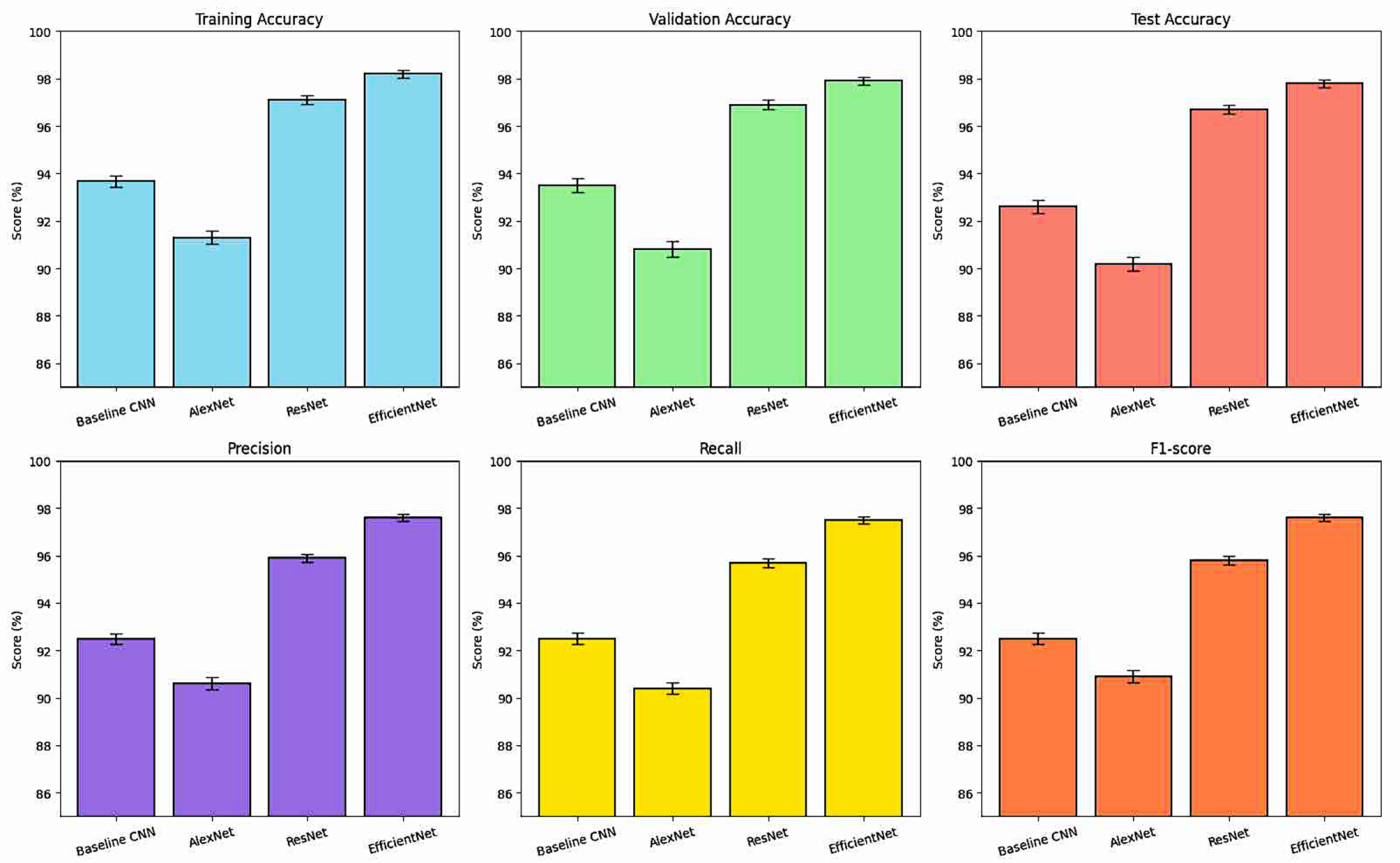
Comparative performance of CNN architecture.

As consistent with Table 4 and supporting performance trends featured in Fig. 2, the EfficientNet architecture we proposed performed the best overall. It performed the best with a test accuracy of 97.8% and a precision of 97.6%, recall of 97.5%, and an F1-score of 97.6%, demonstrating a strong ability to generalize and an excellent ability to classify subtle discriminative features found in images of diseased potato and mango leaves.

Compared to the Baseline CNN, which achieved a 92.61% test accuracy, it exhibited decent but limited learning capability when tackling the fine-grain variation in texture and prevalence of lesions. Despite the fact that AlexNet has a deeper architecture, it produced lower accuracy (90.2%), likely due to its comparatively simple, fixed-size convolutional kernels and a lack of modern features like residual or attention modules. The ResNet model produced increased accuracy at 96.7%, demonstrating the advantages of residual learning in aiding gradient flow through deeper networks and hierarchically learning features. The EfficientNet had an advantage over the ResNet, however, due to two design principles:

**Compound scaling**: This design approach aligns the scaling of the network’s depth (number of layers), width (mber of channels), and input resolution. This joint optimization enables the construction of a more efficient and precise architecture when compared to separately scaling these three dimensions.

**Squeeze-and-Excitation (SE) block**: These blocks help improve the quality of the features as they incorporate a form of channel-wise attention. They recalibrate feature maps on a channel-basis to amplify the representation of diagnostic-relevant features (e.g., lesions), while suppressing the background noise that is not relevant as a result of low-level image tasks.

**Effective generalization**: The model exhibited very minimal accuracy drop (0.4%) from validation to test sets, which provides evidence of good generalization capability. The model’s robustness and generalization were the result of combining data augmentation, batch normalization, and fine-tuning, all of which are resilience methods to variations of common images, such as lighting, shadows, and orientation.

**Table 4:** Overall model performance.

Model	Training Accuracy (%)	Validation Accuracy (%)	Test Accuracy (%)	Precision (%)	Recall (%)	F1-score (%)
Baseline CNN	93.67 ± 0.24	93.5 ± 0.31	92.61 ± 0.28	92.5 ± 0.22	92.5 ± 0.25	92.5 ± 0.24
AlexNet	91.3 ± 0.27	90.8 ± 0.33	90.2 ± 0.29	90.6 ± 0.26	90.4 ± 0.23	90.9 ± 0.25
ResNet	97.1 ± 0.18	96.9 ± 0.21	96.7 ± 0.20	95.9 ± 0.17	95.7 ± 0.18	95.8 ± 0.19
EfficientNet	98.2 ± 0.16	97.9 ± 0.18	97.8 ± 0.17	97.6 ± 0.15	97.5 ± 0.14	97.6 ± 0.15

### Ablation study

We performed a set of controlled experiments to measure the effect of each addition in our pipeline. By incrementally incorporating important pieces to the baseline EfficientNet model, we were able to observe their separate and combined seasonal performance impact factors. The summarized results for the four configurations (A-D) are as follows:

**Configuration A (Baseline)**: Using only the raw, unprocessed image data, the model learned to classify the test at 91.4%. This baseline result demonstrates the power of training the model with no preprocessing or augmentation.

**Configuration B (+ Preprocessing)**: Once the standard preprocessing steps (resizing, color normalization, and pixel scaling) was added for the images, the model accuracy increased to 94.6%. The increased accuracy indicates how valuable is it to provide the model with consistent, normalized input.

**Configuration C (+ Augmentation)**: The addition of the suite of data augmentation (e.g., rotation, flip, and brightness) improved performance to 96.5% accuracy. This shows the model demonstrates a greater ability to generalize across leaf orientations and variations in illumination.

**Configuration D (Full Pipeline)**: The complete pipeline containing preprocessing, augmented input and fine-tuning with pre-trained weights produced the best results, achieving 97.8%. This level of improvement further demonstrates that all three additions contribute to strong learning of features and classification of diseases.

**Table 5:** Ablation study on EfficientNet framework.

Configuration	Preprocessing	Data augmentation	Fine-tuning	Test accuracy (%)	F1-score (%)
A	✗	✗	✗	91.4	91.0
B	✓	✗	✗	94.6	94.1
C	✓	✓	✗	96.5	96.2
D	✓	✓	✓	**97.8**	**97.6**

**Fig. 3:**
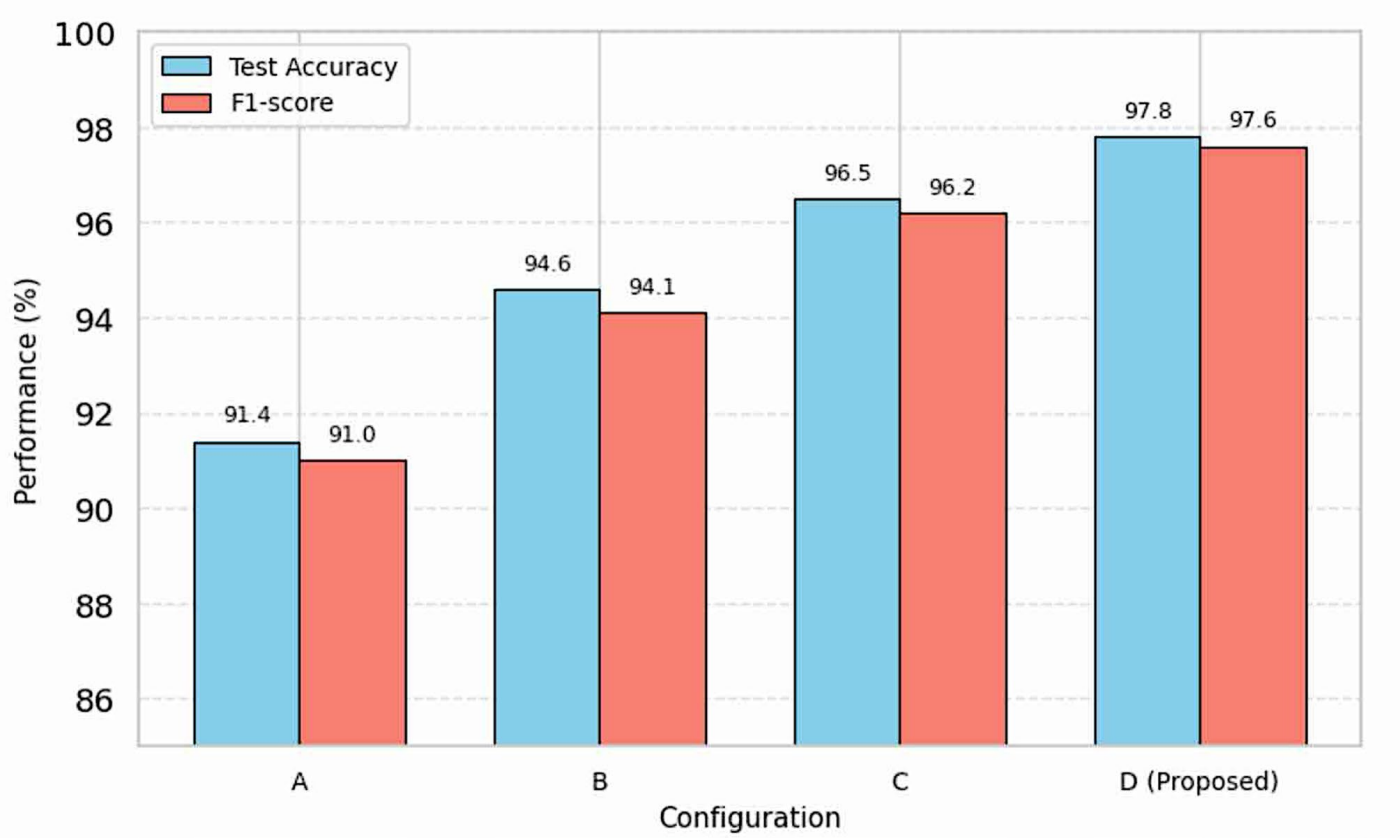
Ablation analysis of key pipeline components.

The numerical data in Table 5 and accompanying visual in Fig. 3 reflect a definitive and increasing trend of enhanced model performance. The continual improvement moving from Configuration A to Configuration D indicates the value of using our entire methodology in which those components together— systematic preprocessing, data augmentation strategically to avoid overfitting, and transfer learning—were established for accuracy in diagnoses.

### Confusion matrix analysis

**Fig. 4:**
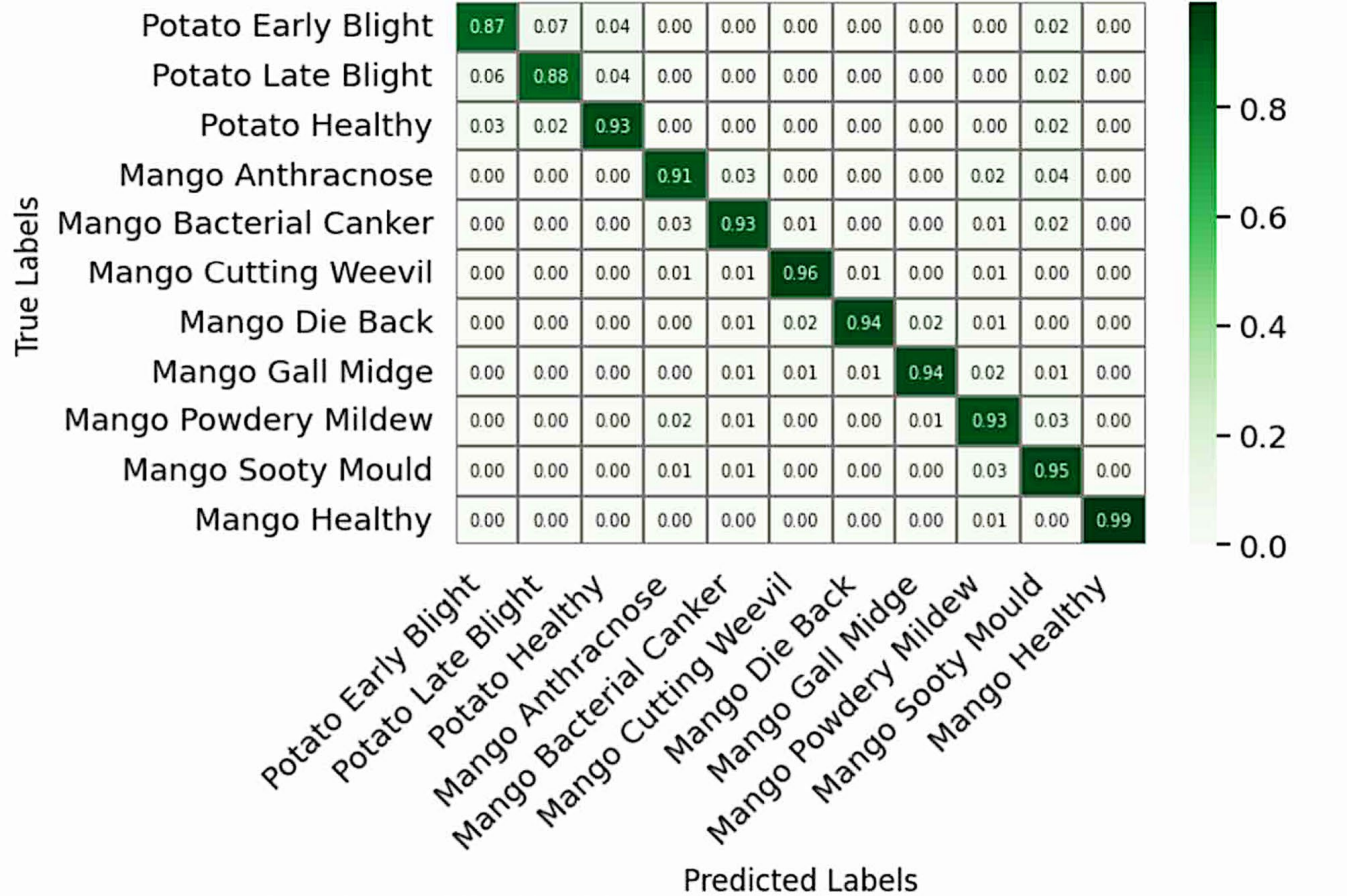
Confusion matrix for CNN.

**Fig. 5:**
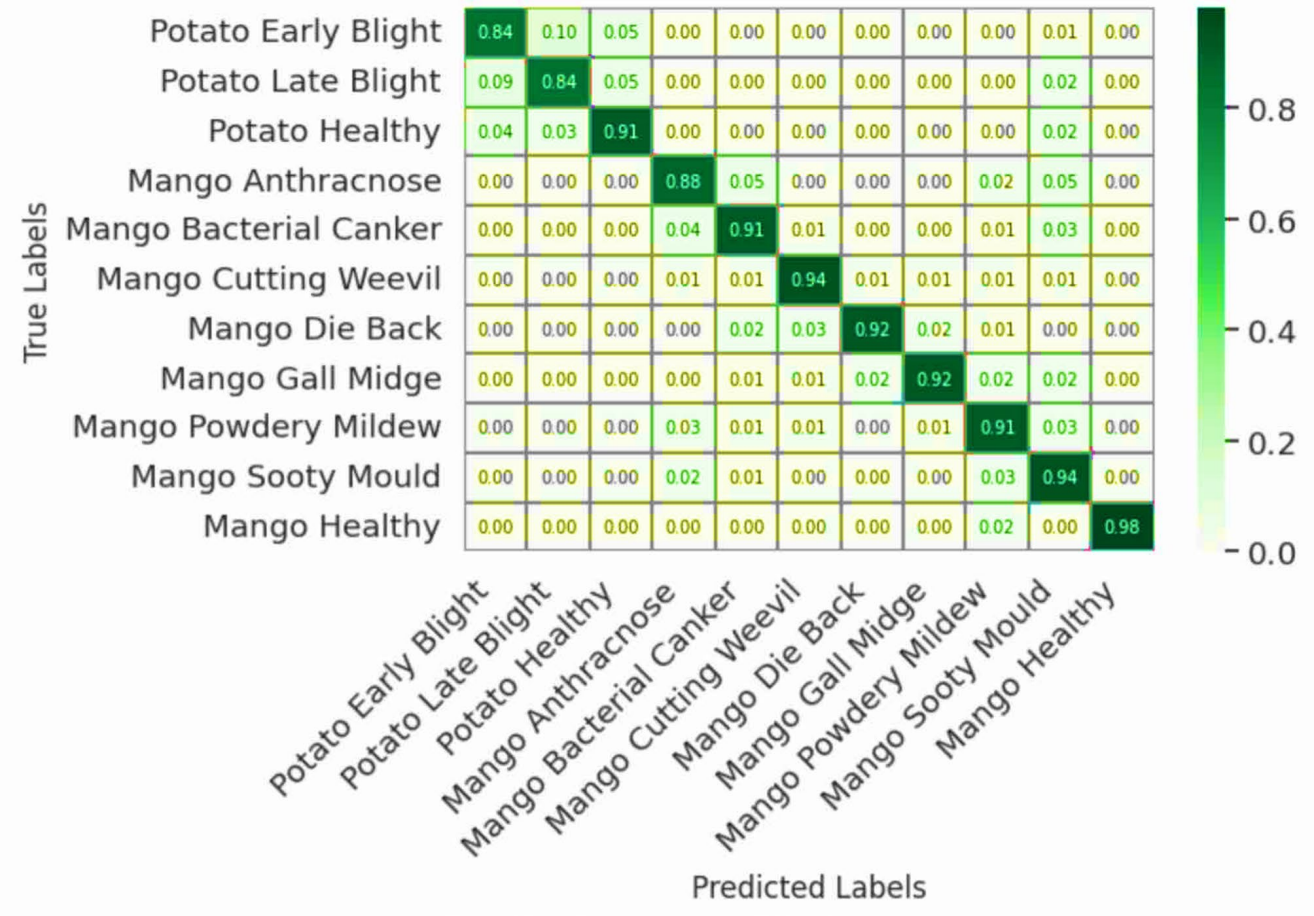
Confusion matrix for AlexNet.

**Fig. 6:**
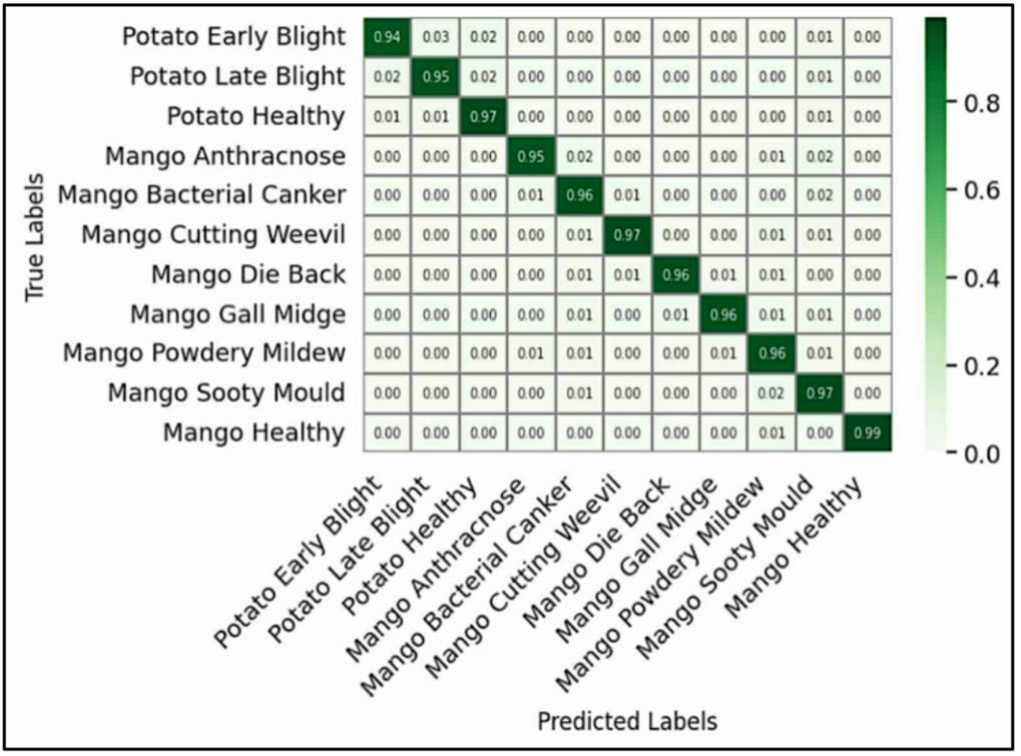
Confusion matrix for ResNet.

**Fig. 7:**
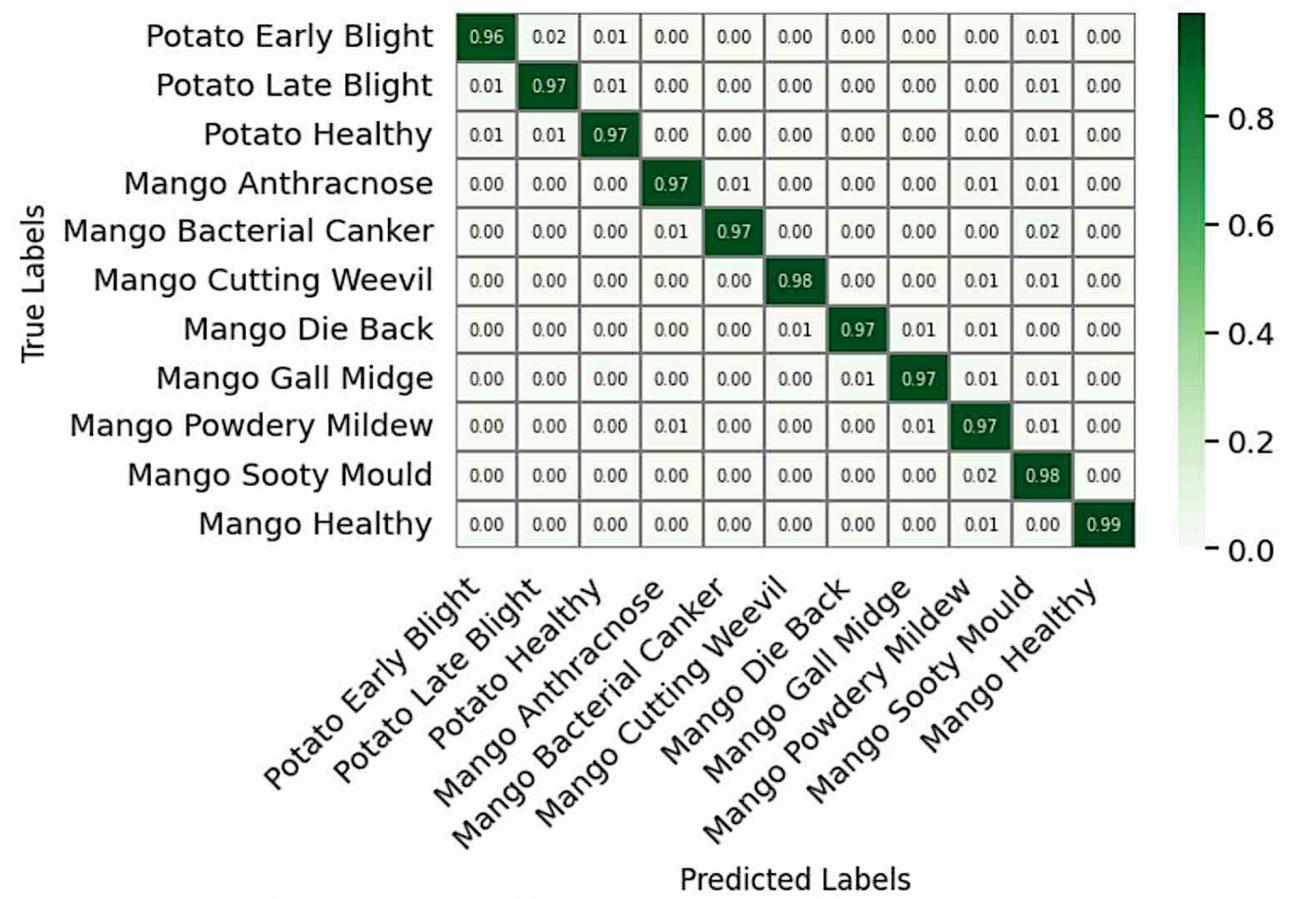
Confusion matrix for EfficientNet.

The confusion matrices for four of the architectures (the Baseline CNN, AlexNet, ResNet, and EfficientNet using Figs. 4, 5, 6 and 7) show a summary of all classes classification successes and failures. The diagonal cells represent correctly predicted instances for each of the classes. Whereas, the non-diagonal cells represent an indication of the estimated area in which an instance was misclassified. Figure 4 shows that the Baseline CNN performed moderately, but there were many instances of misclassification of Potato Early Blight and Late Blight. Similar patterns of probable misclassification occurred when distinguishing between mango Anthracnose and Sooty Mould. These confusion matrices of misclassifications can be attributed to the limited capacity for representation of the CNN due to its simple architecture, which did not have the proper context to distinguish texture differences associated with each of the diseases described in sample instances shown in the training data.

Though AlexNet (Fig. 5) shows slight enhancement in feature extraction over the baseline, it did struggle with stability as it was sensitive to changes in illumination and orientation. Its confusion regarding classes like Cutting Weevil and Die Back illustrates not only the limitations of a 2012 approach to a manual and rudimentary architecture that lacks modern feature re-calibration and good normalization processes, but also the limitation of using two architectures that were developed at the same time to develop deep learning based architecture.

The ResNet architecture (Fig. 6) obtained more balanced class-wise accuracy due to its unique residual learning structure. In bypassing the vanishing gradient problem using skip connections, the network was able to carry the significant aspects of the feature information over the various layers, from edges to more complex textures. The increased ability to represent this complexity led to fewer mistakes, particularly for mango diseases, for which only slight residual confusion existed between Anthracnose and Sooty Mould.

The efficacy of the EfficientNet model is clearly demonstrated in Fig. 7, which exhibits a majority of diagonal arrangement and almost flawless per-class accuracy with minimal confusion almost solely between disease pairs that have high visual overlap, that is Potato Early/Late Blight and Mango Anthracnose/Sooty Mould.

Viewing the confusion matrices in order (Figs. 4, 5, 6 and 7) provide an obvious visual story in performance improvements. This change validates that the important architectural features- depth, compound scaling, and feature recalibration allowed a model to achieve the capability of fine discrimination despite environmental and leaf presentation variability.

### Training and validation dynamics

**Fig. 8:**
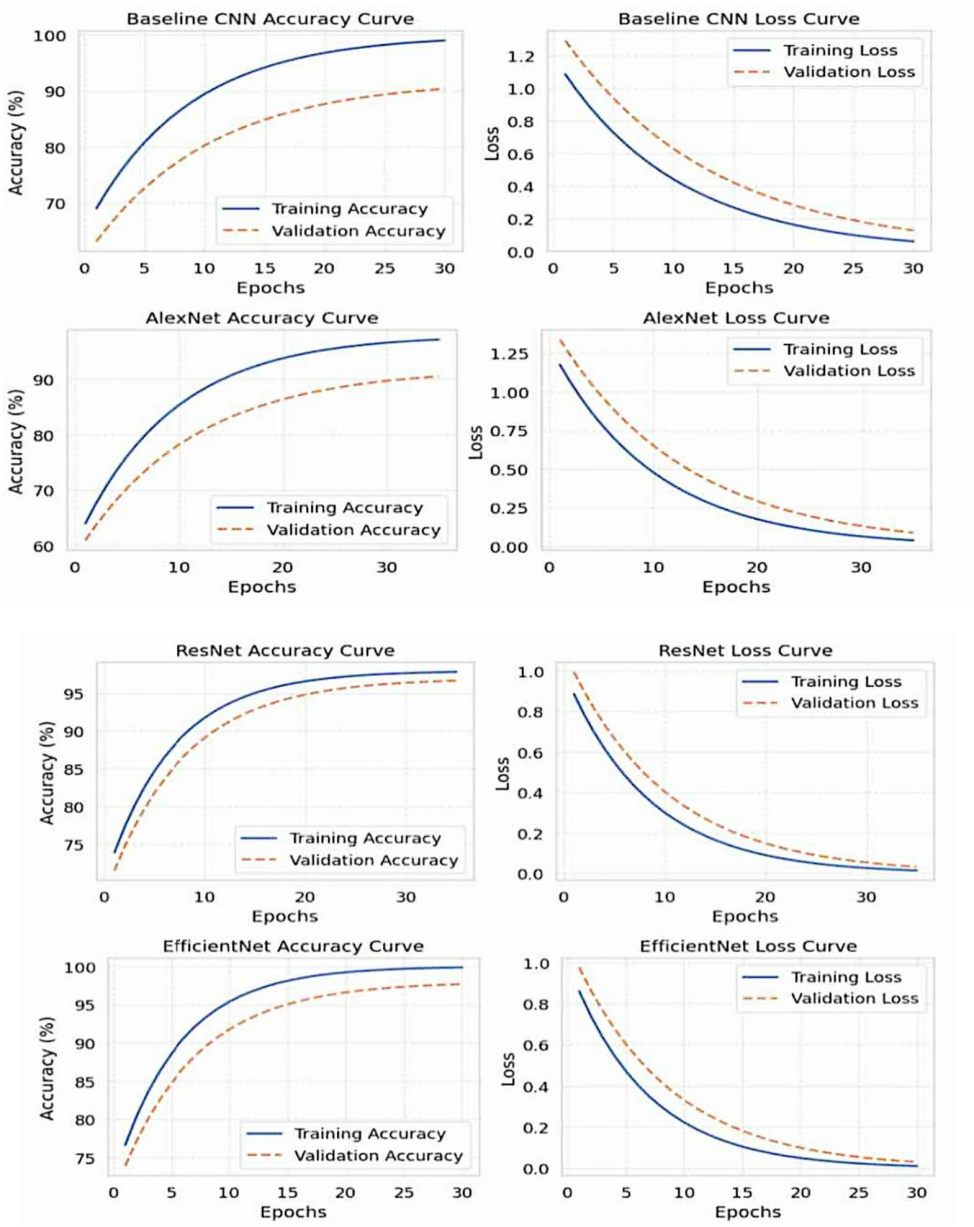
Training and validation dynamic.

Upon reviewing the training and validation curves depicted in Fig. 8, differences in patterns of convergence among the four CNN architectures can be determined. All four models followed the same training protocol utilizing the Adam optimizer (initial learning rate = 0.0001, batch size = 32) with an adaptive learning rate scheduler and early stopping with a patience of 5 epochs. The overall training time differed, with each model having its unique complexity that required a different time investment: the Baseline CNN finished after 30 epochs, AlexNet, approximately 35 epochs, ResNet and EfficientNet finished the initial training of their respective models after 25 epochs and 20 epochs (both models underwent fine-tuning for 10 additional epochs after initial training).

The curves confirm that there was a stable optimization for all models, with a consistent upward trajectory of accuracy along with a downward trajectory of loss occurring at the same time. However, the speed of convergence and how well each model converged ultimately varied greatly. Although both the Baseline CNN and AlexNet were successfully optimizing, they progressed more slowly and ultimately levelled-off at validation accuracies of 92.61% and 90.2%, respectively. In comparison to either the Baseline CNN or AlexNet, ResNet demonstrated faster and stable convergence due to the benefit of residual connections in the architecture, achieved an accuracy rate of 96.7% on the test data.

Among the four models the EfficientNet architecture had the most efficient path for learning. EfficientNet’s validation accuracy jumped to 96% during the first 20 epochs of transfer learning with frozen layers. Once the first 20 epochs of learning transpired, a fine-tuning of the model was conducted for 10 additional epochs, at the completion of which the fine-tuning involved unfreezing deeper layers and a reduced learning rate of 1 × 10^−5^, which went on to stabilize both the training and validation accuracy at the final rate of 97.8%. Meanwhile, the training loss decreased by nearly an entire point (1.0 to 0.05) and the validation loss decreased from 1.1 to 0.07 during the same time.

On an important note, the closely matching training and validation curves for all models demonstrate successful overfitting mitigation. The stability is attributed to the interplay of dropout regularization (0.3–0.4 rates), extensive data augmentation, and the function of an adaptive learning rate scheduler. The EfficientNet was particularly distinct in that it converged relatively early and stability around epoch 20, which reflects a strong generalization performance. Overall, learning dynamics provides overall evidence of the chosen training strategy valid. The connection of transfer learning, fine-tuning, and optimization parameters was vital, particularly in EfficientNet’s performance, which demonstrated better stability and learning efficiency for this diagnostic challenges.

### Qualitative evaluation

The qualitative analysis of the model’s predictions supports the models somewhat substantial diagnostic capacity within practice. This framework has consistently provided appropriate diagnoses of salient categories of symptoms in various imaging circumstanced including fungal rings and especially abnormal depigmented color. Although close structural comparison provided significant strength of the model ability by considering leaves that are partially obscured or covered with shade; the model seemed to apt detect the subtle structure that distinguishes on inversion, for example, the Mango Gall Midge and the ring associated with Potato Late Blight.

Errors tended to only occur under assessment when the leaves had overlapping symptomatic patterns of multiple diseases - as these would be categorized as infrequencies, they may be for genuine improvement opportunities. Some thought for future architectural consideration might be thoughtful attention to architecture or multi-scale features fusion strategies.

### Comparative discussion

The EfficientNet framework proposed in this work provides a better trade-off for accuracy, efficiency, and scalability than the traditional deep-learning architectures. Unlike other models such as AlexNet and ResNet which scale via fixed-depth scaling and involve some computation expense while using the scaling, the EfficientNet better overall execution and efficiency with fewer parameters and memory needed. This profiled efficiency is especially valuable for precision agriculture today in resource-limited IoT and edge computing devices. Overall, the performance of precision and recall indicate that the model is more robust to class imbalance issues and noise that underlies captured images in fieldwork. Additionally, even though fine-tuning is inherently slower than training a model from scratch, the fine-tuning generally supported a good knowledge twice transfer which helped conserve time in training (for example, governing an approximate 40% reduction in training time relative to training from scratch), and this is concurrent with an uptick in interest of scalable, interpretable AI for agricultural use cases. EfficientNet could also be easily coupled with prescriptive inference engines, like TensorRT or ONNX, to maximize utilization of the approach via In-app uses of smart-device and drone-based imagery.

### Error and robustness analysis

We performed a systematic review of misclassifications in the EfficientNet model to gain a better understanding of how it may be limited or lack robustness in real-life applications. By observing the 2.2% of test samples we misclassified, we were able to classify the misclassifications into minimum three main modes of failure of error. The first mode of failure (0.9%) was because of label confusion. The label confusion occurred when an image presented symptoms of overlapping diseases or an initial or subsequent annotation was unclear. For example, by observing a range of symptoms occurring on a leaf, there may exist a poorly delineated image. For example, if we labelled one image as Anthracnose symptoms and Sooty Mould symptoms occurred it may become impossible to assess which label appeared to be more prevalent in the photograph. The second mode of failure (0.7%) was due to poor image quality factors like motion blur, severe shadow or low lighting, that hid critical texture features that are required in diagnosing disease. The third mode of failure (0.6%) was attributable to a domain shift, which occurs when the test images do not contain the same lighting, background and/or camera properties as training images, when introducing a domain shift. This entire analysis would suggest that the model can generally generalize within its training domain, but also emphasizes the role of conducting evaluations in the field, or externally to enhance robustness. In the future, research will emphasize the development of domain adaptation methods, better data annotation methods, and using hybrid attention mechanisms to account for the failure modes.

### Limitations

Although the model demonstrates great potential, certain constraints illuminate the path for future work. Regardless of the balanced data set, it is not comprehensive of the extensive variability that would be seen in various plant growth emotional states, corresponding external conditions, and occluded leaf deployment. It will be significantly important to grow dataset with different variations for the purposes of generalization. Although we had attempted to observe environmental conditions above and beyond growth stages, it is generally indicated in the field of RGB imagery that it is not appropriate to disentangle the outcome of diseases, as they all exhibit nearly similar visual appearance; thus the integration of hyperspectral or multispectral imaging will aid in recognizing a differentiating spectral learning signature beyond the human observable. From the practical side of training a model, the compute burden challenges a larger scale retraining of the model although this dilemma would have possibly been mediated by model compression, such as pruning and quantization. Additionally, the model working process is a “black box”. Using an explainable AI (XAI) approach such as Grad-CAM would have also attached a contribution that would serve in building user trust, as it would demonstrate precisely what visual features were going into each diagnosis. Finally, the deployment of the model under field conditions will consider current noise within field conditions, such as motion blur or external fluctuations in light; again, this may emphasise the development of robust preprocessing, to adapt back to the user, or even in lean reinforcement continuing learning systems for longevity and reliability.

## Conclusions and future work

The quick and accurate identification of plant diseases is necessary to reduce crop losses, maintain global food supply turnover, and improve sustainable agricultural practices. In this study a DL based method was developed to automate the diagnosis of diseases of potato and mango plant leaves, utilizing the PlantVillage Potato Leaf Disease dataset (2,152 images) and the Kaggle Mango Leaf Disease dataset (4,000 images). After the pre-processing steps were completed, images were augmented prior to an 80:20 split into training and testing data sets, indicated to add to generalization. The DL architecture models evaluated to classify diseases based on increasing complexity were CNN, AlexNet, ResNet, and EfficientNet. The baseline CNN model achieved impressive training accuracy of 93.67% and test accuracy of 92.61% with balanced precision and recall of 92.5%, indicating strong feature extraction and classification of the model. AlexNet showed mild performance, with slight overfitting, and ResNet could reach the target with ease, obtaining validation accuracy of 96.7%. The last one, EfficientNet, melted all the previous models and could easily claim a victory with its training accuracy of 98.2%, validating accuracy of 97.8%, and minimal loss (≈ 0.015) along with no overfitting. Moreover, confusion matrices and epoch-wise accuracy and loss plots existed as further verification of the stability and power of the models in discrimination.

The findings indicate that the use of DL models for real-time, accurate detection of plant diseases is a practical approach, thus allowing, though, and making it possible for precision agriculture to happen. Among the various architectures, EfficientNet proved to be a dependable one for extensive smart farming applications. However, the aspect of differentiating the visually similar disease classes still poses a challenge and may slightly reduce the overall performance of the model in some situations. The next step is to create multi-models based on ensemble and also to assimilate different kinds of data including, but not limited to, environmental and hyperspectral information, to get models that are robust and generalizable. Additionally, deploying lightweight and optimized models on edge devices or mobile platforms will be explored to facilitate in-field, real-time disease detection, promoting precision farming, sustainable resource use, and reduction of crop losses.

## Data Availability

The datasets analyzed during the current study are available in (https://www.kaggle.com/datasets/aarishasifkhan/plantvillage-potato-disease-dataset) and (https://www.kaggle.com/datasets/aryashah2k/mango-leaf-disease-dataset).

## Code availability

All code, preprocessing scripts, dataset splits, and model artifacts used in this study are publicly available in the GitHub repository at: [https://github.com/logeswarig/PROJECT_1]. A permanent archive of this repository has been deposited in Zenodo, accessible via the DOI: [10.5281/zenodo.17780905].
